# Household and Environmental Determinants Influencing Atopic Dermatitis Among Young Rural Children in the Ehlanzeni District Municipality

**DOI:** 10.3390/ijerph23020182

**Published:** 2026-01-31

**Authors:** Thokozani P. Mbonane

**Affiliations:** Department of Environmental Health, Faculty of Health Sciences, Doornfontein Campus, University of Johannesburg, Johannesburg 2006, South Africa; tmbonane@uj.ac.za

**Keywords:** atopic dermatitis, household and environmental determinants, children, rural areas, prevalence

## Abstract

**Highlights:**

**Public health relevance—How does this work relate to a public health issue?**

- Atopic dermatitis (AD) remains a prevalent condition among young children in both urban and rural settings.
- Access to primary health services for the management of atopic dermatitis is constrained in rural regions.

**Public health significance—Why is this work of significance to public health?**

- The prevalence of atopic dermatitis in rural areas is comparable to that observed in urban areas, where there is a greater volume of research conducted.
- Exposure to second-hand smoke within the household environment serves as a trigger for atopic dermatitis in rural populations.
- The use of traditional, homemade utensils is a contributing factor to the onset of atopic dermatitis in children residing in rural areas.

**Public health implications—What are the key implications or messages for practitioners, policy makers, and/or researchers in public health?**

- There is a necessity for the implementation of environmental health programs, such as educational campaigns, to facilitate the prevention of AD.

**Abstract:**

Background: This study aimed to determine the prevalence of atopic dermatitis (AD), as well as the determinants that contribute to its development, particularly household and environmental determinants, in young children residing in a rural area in South Africa. There is a lack of scientific evidence regarding the determinants, particularly environmental factors, of AD among children living in rural areas. Therefore, this study aimed to identify the household and environmental determinants influencing atopic dermatitis in young rural children in the Ehlanzeni District Municipality. Methods: A cross-sectional analytical study was conducted, wherein mothers/caregivers and their children were purposefully recruited as participants. Data collection involved the utilization of an adapted version of the International Study of Asthma and Allergies in Childhood questionnaire (ISAAC), which was then analyzed using Stata MP version 18. Result: The study included a total of 881 mothers/caregivers, with a majority of the child participants being boys (n = 477, 54.14%). The prevalence rates of historical and current AD were found to be 13.96% and 18.62%, respectively. Natural birth was found to be a protective factor for both history (Adjusted Odds Ratio [AOR]: 0.094; *p* < 0.001) and current (AOR: 0.261: <0.001) AD. The use of a traditional broom for sweeping the floor, exposure to household environmental tobacco smoking, and residing in a household that has ongoing renovations were associated with both history and current AD. Conclusions: These findings demonstrate that the prevalence of AD in rural areas is high among children. Consequently, there is a need to provide primary health care services, particularly for skin diseases, which are currently limited in rural areas. Additionally, environmental health services could play a crucial role in the management and control (especially proactive programs such as educational campaigns) of AD and similar conditions in rural areas.

## 1. Introduction

Atopic dermatitis (AD) is a common multifactorial chronic skin disease that is influenced by environmental determinants. It affects a significant number of young children worldwide [1,2]. Studies estimate that 15–20% of children globally experience AD each year [3]. In sub-Saharan countries, the prevalence of AD in children ranges from 4.7% to 23% [4]. Scientific evidence suggests that children in urban areas have a higher risk of developing AD compared to those in rural areas [5]. However, there are limited studies conducted in sub-Saharan regions that reported the prevalence of AD among young rural children [6]. It is important to note that children in rural areas may have limited access to healthcare services, including those for skin conditions, due to the shortage or absence of primary healthcare facilities [7,8]. While genetic predisposition plays a role in AD, household and environmental determinants also contribute significantly to the development of this condition [9,10,11].

Current evidence shows that AD can be triggered by numerous individual, genetic, and household (home environment) determinants [12]. Family history of AD, personal hygiene products, sex (male children were at high risk), child diet, food allergies, and history of eczema, allergies, hay fever, or asthma have been linked to AD in different environments [13,14]. Furthermore, AD has been associated with season of birth, method of birth, breastfeeding duration, number of siblings, and ordinal position [15,16,17,18]. A few studies have shown that home/residential determinants such as indoor pet ownership, and household income, play a role in triggering AD.

Numerous environmental determinants contribute or trigger the development of AD among children [10]. These determinants included climatological determinants, air pollution and related activities, house dampness, mold in the walls, and residing near bushes or green areas [19]. A recent analytical cross-sectional study in an urban area conducted among schoolchildren 7 years or younger showed that current AD was positively associated with household environmental tobacco smoke (ETS) exposure, fuel use for cooking and heating, use of building materials (renovation), house with new indoor painting and new furniture, broom stick, indoor dust, and frequency of trucks transit through the neighboring streets [20].

In recent years, several studies have indicated that AD is more common among urban children compared to those living in rural areas [6,21,22]. A community-based study conducted in China discovered that the prevalence of AD was higher in urban areas (10.2%) as opposed to rural areas (4.6%) [22]. However, many of the residential and environmental determinants associated with AD are linked to poor socio-economic conditions, which are often prevalent in rural communities. Therefore, the researcher hypothesized that children living in rural areas might be exposed to household and environmental determinants linked to atopic determinants. The objectives of the study were: (i) to describe the prevalence of AD among rural young children; (ii) to identify household and environmental determinants; and (iii) to assess the determinants that influence the prevalence of AD among young rural children from Louisville, Mpumalanga Province, South Africa.

## 2. Materials and Methods

### 2.1. Study Design and Setting

A cross-sectional analytical study was conducted between August 2021 and September 2023. The study was conducted in a rural residential area (Louisville) of Barberton town in Mpumalanga Province, South Africa. The area is situated near the Lily gold mine and is surrounded by agricultural activities. The main gravel road to access the farms and mine is in this residential area.

### 2.2. Population and Sampling

The study sample consisted of caregivers/mothers and their children residing in a rural residential area. The children were between the ages of 5 and 13, and it was required that the child had resided in the area with their parents/guardian from the age of 12 months old or younger. Children who were born outside the study site were excluded from the study, and child-headed households were not considered. Those who were approached and agreed to participate were enrolled in the study. Participants were enrolled in the study using convenience sampling (chosen to ensure direct access to identified potential participants residing near illegal mining activities); families with children were identified beforehand with the support of the local Ward-based outreach teams (WBOTs), and the research teams visited those households. The WBOTs are government structures that are linked to a local health care facility and are responsible for conducting “community, household, and individual-level health assessments”. These teams normally included community health workers led by a nurse. A register of the visited households was kept during data collection to ensure that there was no duplication or visiting of the same households.

To determine the sample size for the study, the following Formula (1) was used. In this formula, “n” represents the sample size, “Z” is the Z-value corresponding to a 95% confidence level (1.96), “p” is the estimated sample proportion (0.5), and “e” is the margin of error, which was set at 0.05. Thus, the estimated population for the study was 384.

N = [z^2^ × p × (1 − p)]/e^2^. (1)

### 2.3. Data Collection

The data was collected in three different timeframes: August 2021, September 2022, and September 2023. Household identification numbers (local stand numbers) were recorded to prevent visiting the same households multiple times. The data were collected using an adapted and modified International Study of Asthma and Allergies in Childhood (ISAAC) questionnaire (see Supplementary File S1) [23,24,25,26]. The questionnaire has been used in South African studies and elsewhere [20,27,28]. Trained research assistants (fourth-year environmental health students) administered the questionnaire and interviewed mothers/caregivers. During data collection, the research assistants were paired (one research assistant needed to be conversant with either isiSwati or siTsonga). The questionnaire was translated into isiSwati and siTsonga, the two most common languages in Louisville, before data collection and the responses were translated back to English by two professional linguists. The questionnaire was divided into socio-demographics (participant characteristics), study health outcome, and household, dietary, and environmental determinants. The questions on determinants were based on the scientific literature and previous studies in a similar population [10,21,22,29,30,31,32,33,34,35,36,37].

Socio-demographic data included: the mother/caregiver’s educational level (no schooling/primary school/high school), income sources (government social grants/self-employed/salary), income level measured in South African Rands (ZAR) with the 14.6206 = 1 United States Dollars (USD) exchange rate at the time of data collection (equal or less than ZAR 2500/ZAR 2501–ZAR 7500/Above ZAR 7500), child sex (male/female), age (7 years old or younger/8–10 years old/11–13 years old), childbirth method (caesarian/natural birth), the ordinal position of the child (firstborn/middle/lastborn), preschool (remote/local), food allergy (no/yes), and history of AD in the family (no/yes).

#### 2.3.1. Study Health Outcomes

The study’s health outcome variables were based on previous scientific literature and studies conducted in similar areas and aligned to the ISAAC criteria for screening for AD [20,27]. History of AD was determined by the positive (yes) to the following question by the mother/caregiver: Has the child had AD at any time before the last six months? The current AD diagnosis was determined by asking the mothers/guardians the following: (i) has the child experienced a persistent dry and itchy rash, (ii) a rash with inflamed skin, and (iii) persistent dry and itchy rash and inflamed skin in the past 6 months.

#### 2.3.2. Household and Dietary Determinants

Household and dietary determinants of AD included were as follows: having an indoor domestic cat (yes/no), having an outdoor domestic dog (yes/no), using chemicals for cleaning (occasional/every day), using a traditional broom for sweeping the floor (yes/no), having an old mat or rag at home (yes/no), consuming house milked dairy products (yes/no), consuming household hatched eggs and homegrown vegetables and fruits (yes/no), frequency of dietary intake by the child of eggs, homegrown vegetables and fruits and peanuts (seldom/sometimes/regularly), and buying (or replacing) new furniture during the mother’s pregnant period, or when the child was younger than 12 months of age (yes/no).

#### 2.3.3. Environmental Determinants

Environmental determinants were as follows: exposure to environmental tobacco smoking at home (yes/no), location of the household street (back street/away from the busy main road)/main street), house surrounding (bushes/no bushes), cooking and heating fuel (electricity/paraffin/coal), dampness on the inside of the house (yes/no), stagnant water within walking distance of the home (yes/no) and house, any house renovations during the mother’s pregnancy or while the child was younger than 12 months (yes/no).

### 2.4. Data Analysis

The data was analyzed using Stata MP software (version 18). Tables and figures were used to present the findings regarding frequency and percentages. Fischer’s exact test was employed to determine statistically significant differences between variables. The association between the prevalence of AD and socio-demographic variables and household and environmental determinants was examined. Initially, a bivariate regression analysis was conducted, and the variables that demonstrated a statistically significant association (*p*-value of 0.05 or less) with the prevalence of AD were reported as an unadjusted odds ratio (UOR). The final model consisted of a multivariate automated stepwise backwards regression analysis (all the determinants were included in the final model), utilizing statistical variables where a *p*-value of 0.05 or lower was considered statistically significant and reported as an adjusted odds ratio (AOR).

### 2.5. Ethical Approval and Consent to Participate

The study obtained ethical clearance from the University of Johannesburg, Faculty of Health Sciences Research Ethics Committee (REC-01-11-2019). Parents and mothers/caregivers gave their informed consent before data collection.

## 3. Results

### 3.1. Characteristics of the Study Participants

Most of the mothers/caregivers had a high school educational level (n = 360, 41.32%), were dependent on government social grants for survival as the source of income (n = 314, 35.64%), and 443 (50.28%) were living on ZAR 2500 or less per month. This study had more boys (n = 477, 54.14%) than girls (n = 404, 45.86%). Most of the study participants were between the ages of 6–8 (n = 332; 37.68%) and were naturally born (n = 709; 80.48%). Furthermore, most were either a middle child (n = 318; 36.10%) or a lastborn (n = 300; 34.05%) in their family structure (position). There were 275 (31.21%) study participants who lived with someone who was dependent on a government social grant for the source of income. Lastly, 452 (51.31%) lived with a mother who is a smoker. Table 1 shows a detailed description of the characteristics of the study participants.

### 3.2. Prevalence of Current AD and History of AD

**Figure 1:**
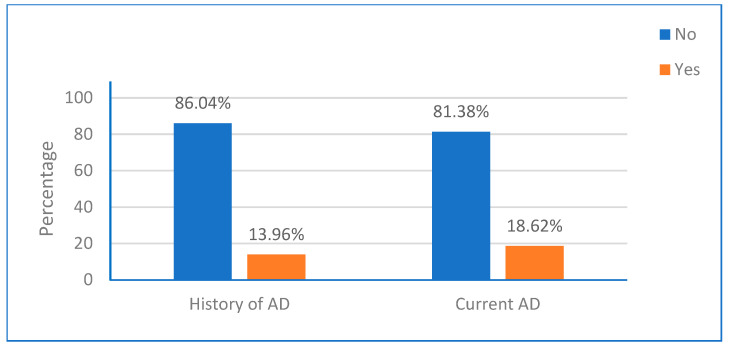
Prevalence of history and current atopic dermatitis.

In the study, 123 children (13.96%) had previously experienced AD. Additionally, 164 children (18.62%) had experienced AD in the past six months, as shown in Figure 1.

### 3.3. Household Determinants of Atopic Dermatitis in the Study

Table 2 shows the distribution of history and current atopic dermatitis (AD) according to household determinants. Among children residing in households with cats, the reported cases of a history of AD were 17.07% (n = 21) out of 358 households that owned a cat, while 55 children (44.72%) with a reported history of AD lived in households with dogs as pets. Furthermore, the number of cases of children with a history of AD from households that used daily cleaning chemicals and traditional brooms were 48.78% (n = 60) and 92.68% (n = 114), respectively. Out of 459 households (52.10%) that owned old mats or rugs, 63 reported having children with a history of AD. The cases with a history of AD were high concerning specific dietary factors: 76.67% (n = 98) among those consuming household eggs, 79.67% (n = 98) among those eating homegrown vegetables and fruits, and 60.16% (n = 74) among those who regularly consumed peanuts. Comparative analysis between children with a history of AD and those without indicated statistically significant differences for the following categorical variables: cats (*p* = 0.001), cleaning chemicals (*p* < 0.001), traditional broom (*p* < 0.001), household eggs (*p* = 0.001), eggs (*p* = 0.001), and furniture (*p* < 0.001). There was a high number of current AD reported among children residing in households that utilize cleaning chemicals daily (n = 82; 50%), employ traditional brooms (n = 117; 71.34%), consume household-produced eggs (n = 132; 80.49%), and regularly consume homegrown vegetables and fruits (n = 132; 80.49%). Additionally, a significant proportion of these children reported regular consumption of peanuts (n = 102; 62.20%). Among children diagnosed with current AD, a statistically significant difference was observed when compared to those without current AD across several household and dietary factors: the presence of cats (*p* = 0.001), daily use of cleaning chemicals (*p* < 0.001), ownership and use of traditional grass brooms (*p* < 0.001), consumption of household eggs (*p* < 0.001), regular egg consumption (*p* < 0.001), intake of homegrown vegetables and fruits (*p* < 0.001), regular vegetable consumption (*p* = 0.010), regular peanut consumption (*p* = 0.025), and the presence of new furniture during pregnancy (*p* < 0.001), see Table 2.

### 3.4. Environmental Determinants of Atopic Dermatitis in the Study

Table 3 shows the descriptive analysis of environmental determinants according to and current cases of atopic dermatitis. There were children with AD in both history (n = 97, 75.78%) and current (n = 124, 96.88%) cases in households with environmental tobacco smoking exposure. There were more children with current AD (n = 77, 18.51%) who stayed in households surrounded by bushes when compared to those with a history of AD (n = 59, 14.18%). Most children with AD come from households that use coal for cooking and heating. In children with a history of AD, 14.86% (n = 70) and 13.80% (n = 93) used coal for cooking and heating, respectively. Of children with current AD, 86 (18.26%) and 121 (17.95%) were from households that use coal for cooking and heating, respectively. The analysis reveals a statistically significant difference between children with a history of atopic dermatitis (AD) and those without several environmental determinants. These determinants include exposure to environmental tobacco smoke (*p* = 0.001), house location (*p* = 0.001), type of cooking fuel used (*p* = 0.007), and residing in a house with renovations (*p* < 0.001). Additionally, significant differences were observed for environmental tobacco smoke exposure (*p* = 0.001), street location of the house (*p* = 0.001), cooking fuel type (*p* < 0.001), and the presence of renovations in the house (*p* < 0.001).

### 3.5. Determinants of Atopic Dermatitis in the Study

The bivariate analyses of determinants that were not associated with history and current atopic dermatitis are presented in the appendices (Table A1, Table A2, Table A3 and Table A4). While in Table 4 and Table 5 present the determinants that had an individual association with the health outcome in the study. In the bivariate analysis, history of AD had a significant association with using cleaning chemicals daily (*p* = 00.001), consuming household eggs (*p* = 0.001), recently changed or bought furniture (*p* = 0.001), the use of a childbirth method (*p* = 0.001), using a traditional broom (*p* = 0.001), ETS (*p* = 0.001), and house renovation (*p* = 0.001).

The bivariate analysis showed that current AD was significantly associated with having a pet cat (*p* = 0.001), using cleaning chemicals daily (*p* = 0.001), consuming household eggs (*p* = 0.001), consuming homegrown vegetables and fruits (*p* = 0.014), recently changed or bought furniture (*p* = 0.001), using a traditional broom (*p* = 0.001), ETS (*p* = 0.001), residing in house surrounded by bushes (*p* = 0.001), and house renovation (*p* = 0.001).

In the final multivariate analysis model (Table 4 and Table 5), being born through caesarean was a protective factor for both children with a history of AD (AOR: 0.094; *p* < 0.001) and current AD (AOR: 0.261, *p* < 0.001). While residing in a household that used a grass broom for cleaning the floor (AOR: 1.106, *p* < 0.001), ETS exposure (AOR: 1.141; *p* < 0.001), and renovated house (AOR: 4.020; *p* < 0.001) were determinants for history of AD. Similar current AD was associated with residing in a household that used a grass broom for cleaning the floor (AOR: 1.113; *p* < 0.001), ETS exposure (AOR: 1.721; <0.001), and renovated house (AOR: 3.998, *p* < 0.001). The multivariate analysis findings of determinants that had no significant association either with a history of or current AD in the study are presented in Appendix B.1 and Appendix B.2, respectively.

## 4. Discussion

**Figure 2:**
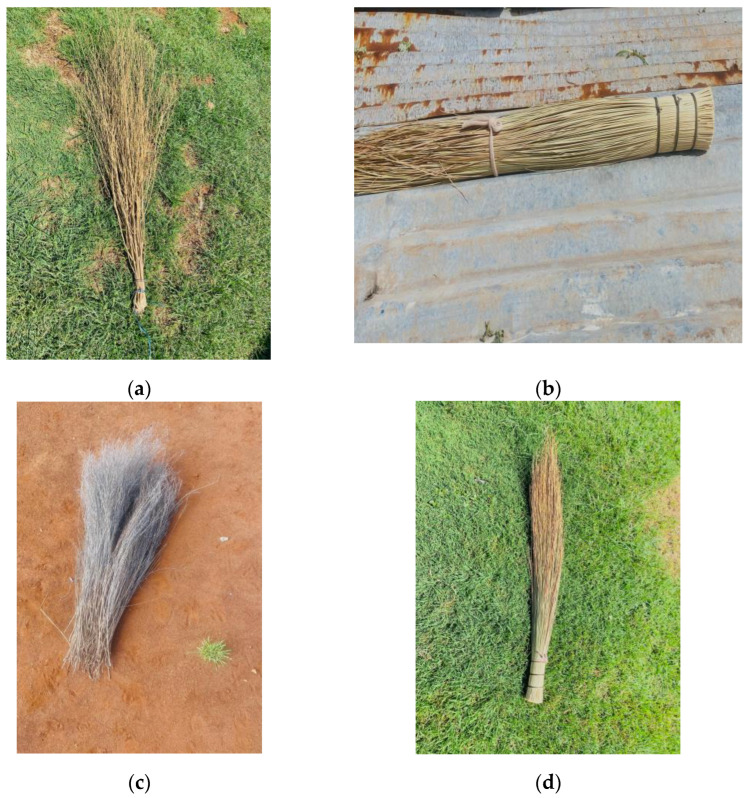
Example of the self-made and informally sold traditional brooms. (**a**) Traditional broom made of tree plant; (**b**) traditional broom made of long strong grass; (**c**) traditional broom made of a different tree plant from figure (**a**); and (**d**) traditional grass broom made of soft long grass.

The study aimed to elucidate the prevalence of AD, both and current, while also assessing determinants, particularly household, dietary, and environmental determinants, associated with the prevalence of AD within the population. The findings indicated the prevalence of both and current AD among the study population. The study revealed that the method of childbirth may significantly influence the development of atopic dermatitis [16,38,39], with natural birth identified as a protective factor. Furthermore, the study revealed a significant positive association between both history and current AD and the utilization of traditional brooms (shown in Figure 2) for floor cleaning, exposure to household environmental tobacco smoke, and experiences of home renovations. Additionally, it was found that the current AD was positively correlated with the presence of an indoor domestic cat. The prevalence of a history of and current atopic dermatitis among children aged 7 to 13 years in rural areas was found to be 13.96% and 18.62%, respectively. This study’s findings fall within the range of prevalence reported in studies conducted in urban areas of South Africa. This similarity may be attributed to the presence of industrial activities, such as agricultural practices and the now-defunct Lily gold mine, close to these rural communities. Previous research has suggested that industrial activities in urban settings may contribute to the onset and progression of atopic dermatitis [6].

Previous studies have reported the role of ETS and house renovation on the development of atopic dermatitis [20,30,40,41,42]. Recently, a South African study in an urban area has shown that there is a positive association between ETS and atopic dermatitis among preschool children aged 7 years [20]. This is similar to other South African studies and elsewhere among young children and adolescents [27,35,36,43,44,45]. The study was in line with previous studies; ETS was associated with history and current atopic dermatitis. The study also shows an association between house renovation during pregnancy or infancy with history and current atopic dermatitis. This finding is consistent with previous studies [19,30]. A cross-sectional study conducted among Chinese children aged 3–6 found that home renovation was a determinant for atopic dermatitis. Both findings are important for parental educational awareness for preventing environmental determinants of atopic dermatitis and similar conditions, especially during pregnancy and infancy.

The utilization of a grass broom to sweep the floor was positively associated with both history and current atopic dermatitis. One potential explanation for this association may be the presence of grass allergens originating from the broom itself, as previous research has indicated that grass pollen can trigger the development of atopic dermatitis [46,47]. Additionally, the broom may serve as a device that captures dust mites and other mites commonly found on the floor daily [48,49]. Nevertheless, further investigation is warranted to determine the underlying cause for the correlation between the use of a grass broom and atopic dermatitis.

The relationship between the childbirth method and the development of atopic dermatitis and other allergic conditions has been a subject of investigation. Although there is no conclusive worldwide evidence, several studies have suggested a potential link between cesarean birth and allergic conditions. For instance, a Swedish National Cohort study involving children aged 5 or younger found that those born by cesarean section were at a higher risk of developing atopic dermatitis in early childhood. These findings align with a Scandinavian cohort study by Hoel et al. in 2023 [38]. Conversely, a cohort study conducted in the United States did not find an association between cesarean section and atopic dermatitis [39]. Similarly, a prospective birth cohort study conducted in Greece also found no association between atopic dermatitis and cesarean-section birth [37]. Notably, the Greek study found that natural birth was a protective factor against atopic dermatitis, which supports the hypothesis that children born through cesarean sections are at risk of developing AD, and natural birth is a protective factor. This is also highlighted in the study findings, which indicate that natural birth is a protective factor. These findings could be valuable for maternal education and encourage expecting mothers to consider natural birth when it is safe, as part of primary prevention for childhood atopic dermatitis.

One of the study’s strengths was using the ISAAC questionnaire, which has been used globally and in local settings. There were two study limitations. The study determined the association using a cross-sectional study and does not show the cause–effect relationship between atopic dermatitis and determinants. Hence, the study recommends a longitudinal study in the same or a similar setting. Lastly, the study did not conduct a clinical diagnosis of atopic dermatitis among participants.

## 5. Conclusions

The study findings show a high prevalence of AD among children in rural areas. The study’s key findings on the determinants of AD, using traditional brooms for floor cleaning, exposure to household environmental tobacco smoke, and experiences of home renovations were found to influence AD in the study, while natural birth was a protective factor. Further investigation is warranted in rural areas regarding the occurrence of atopic dermatitis among children, as these areas often suffer from limited access to healthcare services. Consequently, there is a pressing need to implement environmental prevention programs aimed at safeguarding children residing in rural areas from the triggers of atopic dermatitis.

## Figures and Tables

**Table 1:** Study participants’ characteristics.

Characteristics		Frequency (n)	Percent (%)
Mother/Caregiver’s educational levels	No schooling	144	16.35
	Primary School	201	22.81
	High School	360	41.32
	College/University	176	19.52
Mother/Caregiver’s Income Sources	Government Social Grants	314	35.64
	Self-employed	292	33.14
	Salary	275	31.21
Mother/Caregiver’s income	Equal/less than ZAR 2500	443	50.28
	ZAR 2501–ZAR 7500	233	26.45
	Above ZAR 7500	205	23.27
Mother/Caregiver who is a smoker	No	429	48.70
	Yes	452	51.30
Gender	Female	404	45.86
	Male	477	54.14
Age	5–7 years old	332	37.68
	8–10 years old	290	32.92
	11–13 years old	259	29.40
Childbirth Method	Caesarian	172	19.52
	Natural Birth	709	80.48
Ordinal position	Firstborn	263	29.85
	Middle	318	36.10
	Lastborn	300	34.05
Preschool	Remote	716	81.27
	Local	165	18.73
Food Allergy	No	201	22.81
	Yes	680	77.19
History of AD in the family	No	129	14.64
	Yes	752	85.36

**Table 2:** Household and dietary determinants and atopic dermatitis.

Determinant		Total N (%)	History		*p*-Value ^a^	Total N (%)	Current		*p*-Value
			AD	No AD			AD	No AD	
Cat	No	523 (59.36%)	123 (100%)	400 (52.77%)	0.001	523 (59.36%)	146 (89.02%)	377 (52.58%)	**0.001**
	Yes	358 (40.64%)	0	358 (47.23%)		358 (40.64%)	18 (10.98%)	340 (47.42%)	
Dog	No	478 (54.26%)	68 (55.28%)	410 (54.09%)	0.846	478 (54.26%)	92 (56.10%)	386 (53.84%)	0.664
	Yes	403 (45.91%)	55 (44.72%)	348 (45.91%)		403 (45.74%)	72 (43.90%)	331 (46.16%)	
Cleaning Chemicals	Occasional	632 (71.74%)	63 (51.22%)	569 (75.07%)	**<0.001**	632 (71.74%)	82 (50%)	550 (76.71%)	**<0.001**
	Everyday	249 (28.26%)	60 (48.78%)	189 (24.93%)		249 (28.26%)	82 (50%)	167 (23.29%)	
Broom	No	700 (79.46%)	9 (7.32%)	691 (79.46%)	**<0.001**	700 (79.46%)	47 (28.66%)	653 (91.07%)	**<0.001**
	Yes	181 (20.54)	114 (92.68%)	67 (8.84%)		181 (20.54%)	117 (71.34%)	64 (8.93%)	
Old mats or rugs	No	422 (47.90%)	60 (48.78%)	362 (47.76%)	0.864	422 (47.90%)	89 (54.27%)	333 (46.44%)	0.083
	Yes	459 (52.10%)	63 (51.22%)	396 (52.24%)		459 (52.10%)	75 (45.73%)	384 (53.56%)	
Diary Milk	No	430 (48.81%)	67 (54.47%)	363 (47.89%)	0.206	430 (48.81%)	86 (52.44%)	344 (47.98%)	0.341
	Yes	451 (51.19%)	56 (45.53%)	395 (52.11%)		451 (51.19%)	78 (47.56%)	373 (52.02%)	
Household Eggs	No	748 (84.90%)	25 (20.33%)	723 (95.38%)	**<0.001**	748 (84.90%)	32 (19.51%)	716 (99.86%)	**<0.001**
	Yes	133 (15.10%)	98 (79.67%)	35 (4.62%)		133 (15.10%)	132 (80.49%)	1 (0.14%)	
Eggs	Seldom	195 (22.13%)	18 (14.63%)	177 (23.35%)	**0.001**	195 (22.13%)	19 (11.59%)	176 (24.55%)	**<0.001**
	Sometimes	242 (27.47%)	47 (38.21%)	195 (25.73%)		242 (27.47%)	68 (41.46%)	174 (24.27%)	
	Regular	444 (50.40%)	58 (47.15%)	386 (50.92%)		444 (50.40%)	77 (46.95%)	367 (51.19%)	
Home Grown Vegetable/Fruits	No	179 (2032%)	25 (20.33%)	154 (20.32%)	1.000	179 (20.32%)	32 (19.51%)	147 (20.50%)	0.830
	Yes	702 (79.68%)	98 (79.67%)	604 (79.68%)		702 (79.68%)	132 (80.49%)	570 (79.50%)	
Vegetables	Seldom	158 (17.93%)	22 (17.89%)	136 (17.94%)	0.633	158 (17.93%)	23 (14.02%)	135 (18.83%)	**0.010**
	Sometimes	263 (29.85%)	41 (33.33%)	222 (29.29%)		263 (29.85%)	65 (39.63%)	198 (27.62%)	
	Regular	460 (52.21%)	60 (48.78%)	400 (52.77%)		460 (52.21%)	76 (46.34%)	384 (53.56%)	
Peanuts	Seldom	181 (20.54%)	22 (17.89%)	159 (20.98%)	0.243	181 (20.54%)	30 (18.29%)	151 (21.06%)	0.025
	Sometimes	233 (26.45%)	27 (21.95%)	206 (27.18%)		233 (26.45%)	32 (19.51%)	201 (28.03%)	
	Regular	467 (53.01%)	74 (60.16%)	393 (51.85%)		467 (53.01%)	102 (62.20%)	365 (50.91%)	
Furniture	No	705 (80.02%)	69 (56.10%)	636 (83.91%)	**<0.001**	705 (80.02%)	109 (66.46%)	596 (83.12%)	**<0.001**
	Yes	176 (19.98%)	54 (43.90%)	122 (16.09%)		176 (19.98%)	55 (33.54%)	121 (16.88%)	

^a^ *p*-value was determined using Fischer’s exact test. Bold shows statistical significance at 0.050.

**Table 3:** Environmental determinants in the study.

Environmental Determinants		History		*p*-Value ^a^	Current		*p*-Value
		AD	No AD		AD	No AD	
Environmental Tobacco Smoking Exposure	No	26 (21.14%)	727 (95.91%)	**0.001**	40 (24.39%)	713 (99.44%)	**0.001**
	Yes	97 (78.86%)	31 (4.09%)		124 (75.61%)	4 (0.56%)	
House Street Location	Back Street	8 (6.50%)	758 (100%)	**0.001**	49 (29.88%)	717 (100%)	**0.001**
	Main Street	115 (93.50%)	0		115 (70.12%)	0	
House Surrounding	Bushes	59 (47.97%)	357 (47.10%)	0.922	77 (46.95%)	339 (47.28%)	1.000
	No bushes	64 (52.03%)	401 (52.90%)		87 (53.05%)	378 (52.72%)	
Heating Fuel	Electricity	30 (24.39%)	189 (24.93%)	0.649	43 (26.22%)	176 (24.55%)	0.907
	Paraffin	23 (18.70%)	168 (22.16%)		35 (21.34%)	156 (21.76%)	
	Coal	70 (56.91%)	401 (52.90%)		86 (52.44%)	385 (53.70%)	
Cooking Fuel	Electricity	30 (24.39%)	140 (18.47%)	**0.007**	43 (26.22%)	127 (17.71%)	**<0.001**
	Paraffin	0	37 (4.88%)		0	37 (5.16%)	
	Coal	93 (75.61%)	581 (76.65%)		121 (73.78%)	553 (77.13%)	
Dampness	No	65 (52.85%)	376 (49.60%)	0.560	85 (51.83%)	356 (49.65)	0.665
	Yes	58 (47.15%)	382 (50.40%)		79 (48.17%)	361 (50.35%)	
Stagnant Water	No	63 (51.22%)	359 (47.36%)	0.438	82 (50%)	340 (47.42%)	0.603
	Yes	60 (48.78%)	399 (52.64%)		82 (50%)	377 (52.58%)	
House Renovation	No	98 (79.67%)	284 (37.47%)	**<0.001**	116 (70.73%)	266 (37.10%)	**<0.001**
	Yes	25 (20.33%)	474 (62.53%)		48 (29.27%)	451 (62.90%)	

^a^ *p*-value was determined using Fischer’s exact test. Bold shows statistical significance at 0.050.

**Table 4:** Multilogistic regression analysis of determinants associated with the prevalence of a history of AD.

Determinant		Reference	UOR	Std Error	*p*-Value	95% CI	AOR	Std Error	*p*-Value	95% CI
Childbirth Method	Natural Birth	Caesarian	0.024	0.006	**0.001**	0.240–0.779	0.094	0.042	**<0.001**	0.039–0.224
Broom	Yes	No	1.008	0.003	**0.001**	0.004–1.016	1.106	0.048	**<0.001**	0.044–1.255
ETS	Yes	No	1.311	0.112	**0.001**	0.007–1.020	1.141	0.001	**<0.001**	0.364–1.648
Renovation	Yes	No	6.543	1.546	**0.001**	4.117–10.396	4.020	1.673	**0.001**	1.778–9.088

Bold shows statistical significance at 0.050, while variables that were not statistically significant in the model are presented in the Appendix B.1 (Table A5).

**Table 5:** Multilogistic regression analysis of determinants associated with the prevalence of current AD.

Determinant		Reference	UOR	Std Error	*p*-Value	95% CI	AOR	Std Error	*p*-Value	95% CI
Childbirth Method	Natural Birth	Caesarian	0.352	0.106	**0.001**	0.240–0.779	0.265	0.043	**<0.001**	0.042–0.231
Broom	Yes	No	2.039	0.009	**0.001**	0.026–1.060	1.113	0.049	**<0.001**	1.048–1.265
ETS	Yes	No	1.213	0.133	**0.001**	0.127–1.181	1.721	0.001	**<0.001**	1.642–1.941
Renovation	Yes	No	4.991	1.200	**0.001**	3.265–8.112	3.998	1.601	**0.001**	1.769–9.036

Bold shows statistical significance at 0.050, while variables that were not statistically significant in the model are presented in the Appendix B.2 (Table A6).

## Data Availability

The data can be accessed by the author upon a reasonable request and adhere to the South African Protection of Personal Information Act 4 of 2013 (POPIA).

## Supplementary Materials

The following supporting information can be downloaded at: https://www.mdpi.com/article/10.3390/ijerph23020182/s1. Table S1: Modified ISAAC questionnaire.

## Abbreviations

The following abbreviations are used in this manuscript:

AOR	adjusted odds ratio
AD	Atopic dermatitis
CI	Confidence interval
ETS	Environmental tobacco smoking
ISAAC	International Study of Asthma and Allergies in Childhood Questionnaire
REC	Research Ethics Committee
UOR	Unadjusted odds ratio
ZAR	South African Rands

## Appendix A. Bivariate Analysis

### Appendix A.1

**Table A1:** Bivariate analysis between history of atopic dermatitis and household and dietary determinants.

Household and Dietary Determinants	UOR	Std Error	*p*-Value	95% CI
Cats				
No	Ref			
Yes	1.212	0.232	0.441	0.666–0.711
Dog				
No	Ref			
Yes	1.037	0.180	0.805	0.715–1.539
Cleaning Chemicals				
Occasional	Ref			
Everyday	1.349	0.069	**0.001**	1.236–1.515
Old mats or rugs				
No	Ref			
Yes	1.042	0.203	0.833	0.712–1.525
Diary Milk				
No	Ref			
Yes	1.302	0.254	0.176	0.888–1.908
Household Eggs				
No	Ref			
Yes	2.012	0.003	**0.001**	2.007–2.022
Homegrown Vegetables/Fruits				
No	Ref			
Yes	1.001	0.242	0.998	0.623–1.606
Vegetables				
Seldom	Ref			
Sometimes	0.876	0.250	0.643	0.500–1.534
Regular	1.078	0.289	0.778	0.637–1.825
Peanuts				
Seldom	Ref			
Sometimes	1.056	0.323	0.859	0.580–1.923
Regular	0.735	0.191	0.237	0.441–1.224
Furniture				
No	Ref			
Yes	3.245	0.051	**0.001**	1.163–2.368

Bold means *p*-value was significant at 0.050.

### Appendix A.2

**Table A2:** Bivariate analysis between the history of atopic dermatitis and environmental determinants.

Environmental Determinant	UOR	Std Error	*p*-Value	95% CI
House Street Location				
Back Street	Ref			
Main Street	0.818	0.221	0.200	0.434–0.667
House Surrounding				
Bushes	Ref			
No bushes	1.035	0.202	0.858	0.707–1.516
Cooking Fuel				
Electricity	Ref			
Paraffin	1.159	0.344	0.618	1.648–2.074
Coal	0.909	0.214	0.686	0.573–1.442
Heating Fuel				
Electricity	Ref			
Paraffin	1.212	0.543	0.321	0.432–1.677
Coal	1.339	0.308	0.205	0.853–0.912
Dampness				
No	Ref			
Yes	1.139	0.222	0.505	0.777–1.668
Stagnant Water				
No	Ref			
Yes	1.167	0.227	0.427	0.797–1.709

### Appendix A.3

**Table A3:** Bivariate analysis between current atopic dermatitis and household and dietary determinants.

Household and Dietary Determinants	UOR	Std Error	*p*-Value	95% CI
Cats				
No	Ref			
Yes	7.315	1.908	**0.001**	4.388–12.195
Dog				
No	Ref			
Yes	1.096	0.191	0.600	0.779–1.542
Cleaning Chemicals				
Occasional	Ref			
Everyday	3.304	0.054	**0.001**	0.214–0.432
Old mats or rugs				
No	Ref			
Yes	1.368	0.238	0.051	0.974–1.923
Diary Milk				
No	Ref			
Yes	1.196	0.207	0.303	0.851–1.679
Household Eggs				
No	Ref			
Yes	0.023	0.122	**0.001**	0.133–0.221
Homegrown Vegetable/Fruits				
No	Ref			
Yes	0.940	0.205	0.776	0.614–1.440
Vegetables				
Seldom	Ref			
Sometimes	0.519	0.139	**0.014**	0.307–0.876
Regular	0.861	0.222	0.562	0.519–1.428
Peanuts				
Seldom	Ref			
Sometimes	1.248	0.344	0.422	0.727–2.144
Regular	0.711	0.163	0.137	0.454–1.114
Furniture				
No	Ref			
Yes	1.402	0.078	**0.001**	0.276–058

Bold means *p*-value was significant at 0.050.

### Appendix A.4

**Table A4:** Bivariate analysis between current atopic dermatitis environmental determinants.

Environmental Determinant	UOR	Std Error	*p*-Value	95% CI
House Street Location				
Back Street	Ref			
Main Street	0.330	0.121	0.201	1.232–1.444
House Surrounding				
Bushes	Ref			
No bushes	0.627	0.129	**0.001**	0.015–0.039
Cooking Fuel				
Electricity	Ref			
Paraffin	1.089	0.275	0.739	0.663–1.787
Coal	1.094	0.227	0.666	0.728–1.643
Heating Fuel				
Electricity	Ref			
Paraffin	1.222	0.111	0.107	0.119–0.365
Coal	1.547	0.314	0.031	1.039–2.303
Dampness				
No	Ref			
Yes	1.091	0.189	0.615	0.777–1.532
Stagnant Water				
No	Ref			
Yes	1.109	0.192	0.551	0.790–1.557
House Renovation				
No	Ref			
Yes	4.097	0.771	**0.001**	2.833–5.926

Bold means *p*-value was significant at 0.050.

## Appendix B. Final Model Multivariate Analysis

### Appendix B.1

**Table A5:** Multivariate regression analysis of determinants associated with the prevalence of a history of AD.

Determinants	AOR	Std Error	*p*-Value	95% CI
Cats				
No	Ref			
Yes	1.831	1.262	0.557	0.448–1.541
Dog				
No	Ref			
Yes	1.902	1.283	0.743	0.488–1.668
Cleaning Chemicals				
Occasional	Ref			
Everyday	1.532	1.167	0.287	1.045–1.986
Old mats or rugs				
No	Ref			
Yes	1.791	1.247	0.453	0.428–1.460
Diary Milk				
No	Ref			
Yes	1.400	0.252	0.145	0.116–1.372
Household Eggs				
No	Ref			
Yes	2.007	1.653	0.177	1.211–1.891
Homegrown Vegetables/Fruits				
No	Ref			
Yes	1.702	0.292	0.395	0.311–1.586
Vegetables				
Seldom	Ref			
Sometimes	0.935	0.030	0.215	0.682–1.492
Regular	1.401	0.695	0.497	0.530–1.705
Peanuts				
Seldom	Ref			
Sometimes	1.842	1.449	0.748	0.296–1.396
Regular	1.034	0.504	0.946	0.397–1.689
Furniture				
No	Ref			
Yes	2.116	0.044	1.101	1.055–1.24
House Street Location				
Back Street	Ref			
Main Street	1.807	0.309	0.576	0.381–1.711
House Surrounding				
Bushes	Ref			
No bushes	1.096	0.507	0.997	0.443–1.712
Cooking Fuel				
Electricity	Ref			
Paraffin	1.592	0.652	0.285	0.689–1.541
Coal	1.430	0.597	0.392	0.631–1.243
Heating Fuel				
Electricity	Ref			
Paraffin	1.688	0.889	0.324	0.596–1.780
Coal	1.698	0.972	0.355	0.553–1.213
Dampness				
No	Ref			
Yes	1.251	0.410	0.495	01.658–2.378
Stagnant Water				
No	Ref			
Yes	1.608	0.554	0.168	0.819–2.158

### Appendix B.2

**Table A6:** Multivariate regression analysis of determinants associated with the prevalence of current atopic dermatitis.

Household and Dietary Determinants	UOR	Std Error	*p*-Value	95% CI
Cats				
No	Ref			
Yes	3.834	1.370	0.683	1.349–3.992
Dog				
No	Ref			
Yes	1.939	1.402	0.884	0.406–1.273
Cleaning Chemicals				
Occasional	Ref			
Everyday	1.236	1.100	0.277	1.103–1.544
Old mats or rugs				
No	Ref			
Yes	1.444	0.629	0.399	0.615–1.393
Diary Milk				
No	Ref			
Yes	1.155	0.150	0.504	1.023–1.340
Household Eggs				
No	Ref			
Yes	0.348	0.564	0.135	0.012–0.388
Homegrown Vegetables/Fruits				
No	Ref			
Yes	0.940	0.205	0.776	0.614–1.440
Vegetables				
Seldom	Ref			
Sometimes	0.369	0.243	0.130	0.102–1.340
Regular	0.444	0.280	0.199	0.129–1.531
Peanuts				
Seldom	Ref			
Sometimes	1.968	1.273	0.295	0.554–1.994
Regular	0.972	0.550	0.960	0.321–2.947
Furniture				
No	Ref			
Yes	1.201	0.196	0.843	0.070–0.513
House Street Location				
Back Street	Ref			
Main Street	0.605	0.291	0.297	1.236–1.55
House Surrounding				
Bushes	Ref			
No bushes	0.555	0.363	0.368	0.154–1.001
Cooking Fuel				
Electricity	Ref			
Paraffin	1.774	1.390	0.464	1.382–2.240
Coal	1.430	1.014	0.614	1.356–1.738
Heating Fuel				
Electricity	Ref			
Paraffin	1.902	1.053	0.245	1.643–2.628
Coal	1.769	0.970	0.298	1.604–2.181
Dampness				
No	Ref			
Yes	1.474	0.639	0.369	0.632–1.434
Stagnant Water				
No	Ref			
Yes	1.539	0.663	0.317	0.661–1.581
