# Supplementary material for: Household and Environmental Determinants Influencing Atopic Dermatitis Among Young Rural Children in the Ehlanzeni District Municipality
Source: Int J Environ Res Public Health. 2026 Jan 31;23(2):182. doi: 10.3390/ijerph23020182 (PMC12940809; doi:10.3390/ijerph23020182)
Supplement: Supplementary file 1 [file ijerph-23-00182-s001.zip › ijerph-4106433-supplementary.pdf]

Supplementary File S1

S1 Modified ISAAC questionnaire

A. Socio-demographic section

Question	Response
1. What is your educational status?	
	No schooling
	Primary school
	High school
2. What is the main household income source?	
	Government social grants
	Self-employed
	Salary
3. What is the household monthly income?	
	Equal or less than ZAR 2500
	ZA R2501–R7500
	Above ZAR 7500
4. What is your child's gender?	
	Female
	Male
5. What is your child′s age?	
	7 years old or younger
	8–10 years old
	11–13 years old
6. How was your child born (childbirth method)?	
	Caesarian
	Natural birth
7. What is the ordinal position of your child?	
	Firstborn
	Middle
	Lastborn
8. Where did the child attend preschool?	
	Remote (elsewhere)
	Local
9. Does the child have any food allergies?	
	No
	Yes
10. Did anyone in the family have atopic dermatitis or dermatitis?	
	No
	Yes

B. Socio-demographic section

11. Has your child previously (excluding the past six months) had atopic dermatitis?	
	No

	Yes
12. Has your child experienced a persistent dry and itchy rash in the past 6 months?	
	Yes
	No
13. Has your child experienced a rash with inflamed skin in the past 6 months?	
	No
	Yes
14. Has your child experienced a persistent dry and itchy rash and inflamed skin in the past 6 months?	
	No
	Yes

C. Household and Dietary Determinants section

Question	Response
15. Do you have an indoor domestic pet cat in your household?	
	No
	Yes
16. Do you have an outdoor dog as a pet in your household?	
	No
	Yes
17. Do you use chemicals for cleaning such a detergent in your household?	
	No
	Yes
18. Do you use the grass broom to sweep the floor in your household?	
	No
	Yes
19. Do you have an old mat or rag in your household?	
	No
	Yes
20. Does your child drink/consume housed milked dairy products?	
	No
	Yes
21. Does your child eat household hatched eggs?	
	No
	Yes
22. Does your child eat homegrown vegetables and fruits?	
	No
	Yes
23. How often does your child eat homegrown vegetables and fruits?	
	Seldom
	Sometimes
	Regularly
24. Does your child eat peanuts?	
	No
	Yes

25. How often does your child eat peanuts?	
	Seldom
	Sometimes
	Regularly
26. Did you buy new (or replace) furniture during the mother's pregnancy or while the child was younger than 12 months?	
	No
	Yes

D. Environmental Determinants section

Question	Response
27. Does anyone smoke inside the house?	
	No
	Yes
28. Where is the location of the household street?	
	Back road (away from the main street)
	Main street
29. How would you classify the house's surroundings?	
	Bushes
	No bushes
30. What is the most used fuel for heating in the household?	
	Electricity
	Paraffin
	Coal
31. What is the most used fuel for cooking in the household?	
	Electricity
	Paraffin
	Coal
32. Have you seen dampness on the inside of the house?	
	No
	Yes
33. Is there stagnant water within walking distance of the home?	
	No
	Yes
34. Did you have any house renovations during the mother's pregnancy or 12 months after the child was born?	
	No
	Yes
